# *N*-Phenyl-4-(8-phenyl-4,5-dihydro-1,2-benzoxazolo[4,5-*d*]thia­zol-2-yl)piperidine-1-carboxamide

**DOI:** 10.1107/S1600536809021023

**Published:** 2009-06-17

**Authors:** De-Jin Hu, Ming Liu, Tong-Hui Huang, Hai-Yang Tu, Ai-dong Zhang

**Affiliations:** a Key Laboratory of Pesticide & Chemical Biology, College of Chemistry, Central China Normal University, Wuhan 430079, People’s Republic of China

## Abstract

In the title molecule, C_26_H_24_N_4_O_2_S, the dihedral angle between the isoxazole ring and the adjoining benzene ring is 21.4 (5)°, and between the isoxazole ring and the thia­zole ring is 14.3 (4)°. The piperidine ring is in a chair conformation. In the crystal structure, mol­ecules are linked by inter­molecular N—H⋯O and weak C—H⋯O hydrogen bonds into one-dimensional chains along [001].

## Related literature

The title compound is a potential D1 protease inhibitor. D1 protease is a potential herbicidal target, see: Duff *et al.* (2007 ▶). For synthetic details, see: Bond *et al.* (2003 ▶); Hu *et al.* (2009 ▶).
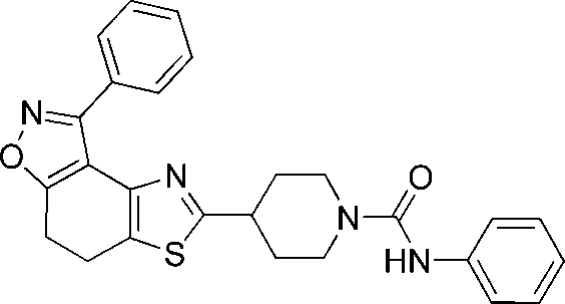


## Experimental

### 

#### Crystal data

C_26_H_24_N_4_O_2_S
*M* *_r_* = 456.55
Monoclinic, 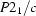

*a* = 22.2844 (6) Å
*b* = 10.1911 (3) Å
*c* = 10.2842 (3) Å
β = 102.282 (2)°
*V* = 2282.11 (11) Å^3^
*Z* = 4
Mo *K*α radiation
μ = 0.17 mm^−1^
*T* = 298 K
0.20 × 0.10 × 0.10 mm

#### Data collection

Bruker SMART CCD diffractometer
Absorption correction: multi-scan (*SADABS*; Sheldrick, 1996 ▶) *T*_min_ = 0.966, *T*_max_ = 0.983
16337 measured reflections
4477 independent reflections
3399 reflections with *I* > 2σ(*I*)
*R*_int_ = 0.093

#### Refinement

*R*[*F* ^2^ > 2σ(*F* ^2^)] = 0.059
*wR*(*F* ^2^) = 0.143
*S* = 1.04
4477 reflections
301 parameters
1 restraint
H atoms treated by a mixture of independent and constrained refinement
Δρ_max_ = 0.24 e Å^−3^
Δρ_min_ = −0.32 e Å^−3^

### 

Data collection: *SMART* (Bruker, 2001 ▶); cell refinement: *SAINT* (Bruker, 2001 ▶); data reduction: *SAINT*; program(s) used to solve structure: *SHELXS97* (Sheldrick, 2008 ▶); program(s) used to refine structure: *SHELXL97* (Sheldrick, 2008 ▶); molecular graphics: *PLATON* (Spek, 2009 ▶); software used to prepare material for publication: *SHELXTL* (Sheldrick, 2008 ▶).

## Supplementary Material

Crystal structure: contains datablocks I, global. DOI: 10.1107/S1600536809021023/lh2833sup1.cif

Structure factors: contains datablocks I. DOI: 10.1107/S1600536809021023/lh2833Isup2.hkl

Additional supplementary materials:  crystallographic information; 3D view; checkCIF report

## Figures and Tables

**Table 1:** Hydrogen-bond geometry (Å, °)

*D*—H⋯*A*	*D*—H	H⋯*A*	*D*⋯*A*	*D*—H⋯*A*
C17—H17*A*⋯O2^i^	0.97	2.40	3.353 (3)	167
N4—H4*A*⋯O2^i^	0.854 (10)	2.145 (12)	2.976 (2)	164 (2)

Symmetry code: (i) 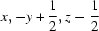.

**Fig. 1.**
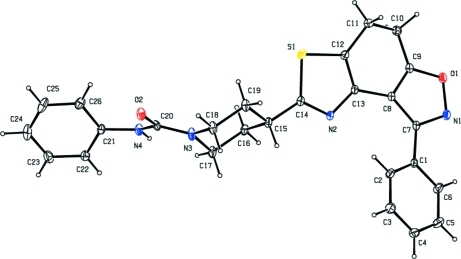
The molecular structure of (I) with displacement ellipsoids drawn at the 10% probability level.

**Fig. 2.**
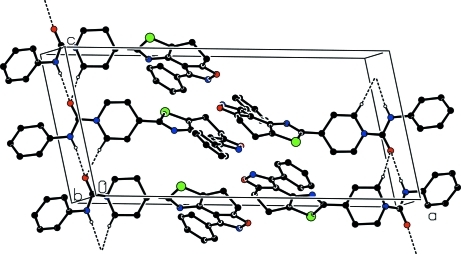
Part of the crystal structure of the title compound with hydrogen bonds drawn as dashed lines. Only H atom involved in hydrogen bonds have been shown.

## supplementary crystallographic information

### Comment

We are interested in the title compound as a potential D1 protease inhibitor. D1 protease is a potential herbicidal target (Duff *et al.* 2007). To find the possible D1 inhibitors, virtual screening was performed and a molecule containing isoxazole, thiazole and piperidine rings was designed and synthesized (Hu *et al.* 2009).

The title moleclue (Fig. 1) contains isoxazole, thiazole, piperdine and two benzene rings. The dihedral angle between the the isoxazole ring and the adjoining benzene ring is 21.4 (5)° and the dihedral angle between the isoxazole and the thiazole rings is 14.3 (4)°. The piperidine ring is in a chair conformation. In the crystal structure, molecules are linked by intermolecular N-H···O and weak C-H···O hydrogen bonds into one-dimensional chains along [001] (see Fig. 2).

### Experimental

3-phenyl-6,7-dihydrobenzo[*d*]isoxazole-4(5*H*)-one was synthesized by a literature method (Bond *et al.*,  2003). This intermediate (1 mmol) was treated with NBS (2.5 mmol) and NH_4_OAc (0.1 mmol) in dry ether to obtain the monobromo ketone and a trace amounts of polybromonated derivatives. The target product was formed by a published procedure (Hu *et al.*, 2009). Slow diffusion of hexane into a ethyl acetate solution of the title compound gave single crystals suitable for X-ray analysis.

### Refinement

All H atoms bonded to C atoms were placed in geometrically idealized positions and included in a riding-model approximation with C—H = 0.93 Å (aromatic), 0.97Å (methylene) and 0.98Å (methine), with *U*_iso_(H) = 1.2*U*_eq_(C). The H atom bonded to N4 was found in a difference Fourier map and refined with the restraint of N—H = 0.86 (2)Å and *U*_iso_(H) = 1.2*U*_eq_(N).

### Crystal data

**Table:**

C_26_H_24_N_4_O_2_S	*F*(000) = 960
*M**_r_* = 456.55	*D*_x_ = 1.329 Mg m^−^^3^
Monoclinic, *P*2_1_/*c*	Mo *K*α radiation, λ = 0.71073 Å
Hall symbol: -P 2ybc	Cell parameters from 4151 reflections
*a* = 22.2844 (6) Å	θ = 2.4–24.8°
*b* = 10.1911 (3) Å	µ = 0.17 mm^−^^1^
*c* = 10.2842 (3) Å	*T* = 298 K
β = 102.282 (2)°	Block, colorless
*V* = 2282.11 (11) Å^3^	0.20 × 0.10 × 0.10 mm
*Z* = 4	

### Data collection

**Table:**

Bruker SMART CCD diffractometer	4477 independent reflections
Radiation source: fine-focus sealed tube	3399 reflections with *I* > 2σ(*I*)
graphite	*R*_int_ = 0.093
φ and ω scans	θ_max_ = 26.0°, θ_min_ = 1.9°
Absorption correction: multi-scan (*SADABS*; Sheldrick, 1996)	*h* = −22→27
*T*_min_ = 0.966, *T*_max_ = 0.983	*k* = −12→12
16337 measured reflections	*l* = −12→12

### Refinement

**Table:**

Refinement on *F*^2^	Primary atom site location: structure-invariant direct methods
Least-squares matrix: full	Secondary atom site location: difference Fourier map
*R*[*F*^2^ > 2σ(*F*^2^)] = 0.059	Hydrogen site location: inferred from neighbouring sites
*wR*(*F*^2^) = 0.143	H atoms treated by a mixture of independent and constrained refinement
*S* = 1.04	*w* = 1/[σ^2^(*F*_o_^2^) + (0.0715*P*)^2^ + 0.1065*P*] where *P* = (*F*_o_^2^ + 2*F*_c_^2^)/3
4477 reflections	(Δ/σ)_max_ = 0.001
301 parameters	Δρ_max_ = 0.24 e Å^−^^3^
1 restraint	Δρ_min_ = −0.31 e Å^−^^3^

### Special details

**Table:**

Geometry. All e.s.d.'s (except the e.s.d. in the dihedral angle between two l.s. planes) are estimated using the full covariance matrix. The cell e.s.d.'s are taken into account individually in the estimation of e.s.d.'s in distances, angles and torsion angles; correlations between e.s.d.'s in cell parameters are only used when they are defined by crystal symmetry. An approximate (isotropic) treatment of cell e.s.d.'s is used for estimating e.s.d.'s involving l.s. planes.
Refinement. Refinement of *F*^2^ against ALL reflections. The weighted *R*-factor *wR* and goodness of fit *S* are based on *F*^2^, conventional *R*-factors *R* are based on *F*, with *F* set to zero for negative *F*^2^. The threshold expression of *F*^2^ > σ(*F*^2^) is used only for calculating *R*-factors(gt) *etc*. and is not relevant to the choice of reflections for refinement. *R*-factors based on *F*^2^ are statistically about twice as large as those based on *F*, and *R*- factors based on ALL data will be even larger.

### Fractional atomic coordinates and isotropic or equivalent isotropic displacement parameters (Å^2^)

**Table:**

	*x*	*y*	*z*	*U*_iso_*/*U*_eq_	
C1	0.38381 (9)	−0.2002 (2)	0.3431 (2)	0.0421 (5)	
C2	0.34151 (12)	−0.2361 (2)	0.4168 (3)	0.0602 (7)	
H2	0.3287	−0.1750	0.4723	0.072*	
C3	0.31805 (13)	−0.3619 (3)	0.4091 (3)	0.0733 (8)	
H3	0.2894	−0.3846	0.4592	0.088*	
C4	0.33650 (12)	−0.4538 (2)	0.3283 (3)	0.0663 (7)	
H4	0.3203	−0.5382	0.3227	0.080*	
C5	0.37882 (15)	−0.4196 (3)	0.2565 (3)	0.0717 (8)	
H5	0.3920	−0.4819	0.2027	0.086*	
C6	0.40231 (12)	−0.2945 (2)	0.2623 (2)	0.0605 (7)	
H6	0.4309	−0.2728	0.2117	0.073*	
C7	0.41255 (9)	−0.0693 (2)	0.35423 (19)	0.0398 (5)	
C8	0.39483 (9)	0.05256 (19)	0.40386 (19)	0.0384 (5)	
C9	0.44192 (9)	0.1341 (2)	0.4002 (2)	0.0424 (5)	
C10	0.44851 (11)	0.2734 (2)	0.4429 (2)	0.0520 (6)	
H10A	0.4326	0.3303	0.3679	0.062*	
H10B	0.4915	0.2941	0.4759	0.062*	
C11	0.41283 (11)	0.2956 (2)	0.5528 (2)	0.0528 (6)	
H11A	0.4376	0.2671	0.6374	0.063*	
H11B	0.4046	0.3885	0.5595	0.063*	
C12	0.35307 (10)	0.2212 (2)	0.5246 (2)	0.0453 (5)	
C13	0.34382 (9)	0.10585 (19)	0.45761 (19)	0.0388 (5)	
C14	0.25309 (10)	0.1164 (2)	0.5099 (2)	0.0449 (5)	
C15	0.18845 (10)	0.0794 (2)	0.5137 (2)	0.0475 (5)	
H15	0.1860	−0.0166	0.5104	0.057*	
C16	0.14331 (10)	0.1320 (2)	0.3913 (2)	0.0491 (6)	
H16A	0.1463	0.2269	0.3893	0.059*	
H16B	0.1543	0.0979	0.3114	0.059*	
C17	0.07811 (10)	0.0935 (3)	0.3925 (2)	0.0556 (6)	
H17A	0.0505	0.1317	0.3161	0.067*	
H17B	0.0740	−0.0012	0.3863	0.067*	
C18	0.10164 (10)	0.0865 (3)	0.6342 (2)	0.0595 (7)	
H18A	0.0978	−0.0083	0.6347	0.071*	
H18B	0.0891	0.1204	0.7125	0.071*	
C19	0.16795 (10)	0.1235 (3)	0.6394 (2)	0.0553 (6)	
H19A	0.1940	0.0830	0.7165	0.066*	
H19B	0.1725	0.2179	0.6487	0.066*	
C20	0.01279 (9)	0.2148 (2)	0.5258 (2)	0.0433 (5)	
C21	−0.07915 (10)	0.3251 (2)	0.4003 (2)	0.0431 (5)	
C22	−0.12464 (11)	0.2917 (2)	0.2914 (2)	0.0561 (6)	
H22	−0.1158	0.2343	0.2277	0.067*	
C23	−0.18266 (12)	0.3426 (3)	0.2767 (3)	0.0740 (8)	
H23	−0.2128	0.3189	0.2031	0.089*	
C24	−0.19687 (15)	0.4266 (3)	0.3673 (4)	0.0837 (10)	
H24	−0.2366	0.4587	0.3577	0.100*	
C25	−0.15178 (17)	0.4637 (3)	0.4735 (3)	0.0849 (10)	
H25	−0.1610	0.5228	0.5353	0.102*	
C26	−0.09237 (13)	0.4143 (3)	0.4903 (2)	0.0650 (7)	
H26	−0.0619	0.4414	0.5617	0.078*	
N1	0.46608 (8)	−0.05766 (18)	0.32118 (18)	0.0496 (5)	
N2	0.28756 (7)	0.04708 (17)	0.44870 (17)	0.0424 (4)	
N3	0.06154 (8)	0.1393 (2)	0.51501 (17)	0.0559 (5)	
N4	−0.02020 (9)	0.2682 (2)	0.41087 (17)	0.0505 (5)	
H4A	−0.0118 (11)	0.252 (2)	0.3352 (14)	0.061*	
O1	0.48560 (7)	0.07301 (15)	0.35047 (15)	0.0512 (4)	
O2	−0.00073 (7)	0.23519 (17)	0.63341 (15)	0.0570 (5)	
S1	0.28825 (3)	0.25928 (6)	0.58254 (6)	0.0556 (2)	

### Atomic displacement parameters (Å^2^)

**Table:**

	*U*^11^	*U*^22^	*U*^33^	*U*^12^	*U*^13^	*U*^23^
C1	0.0326 (11)	0.0463 (12)	0.0465 (12)	0.0101 (9)	0.0064 (9)	−0.0035 (9)
C2	0.0577 (15)	0.0463 (14)	0.0844 (18)	0.0019 (11)	0.0329 (14)	−0.0132 (12)
C3	0.0666 (18)	0.0546 (16)	0.107 (2)	−0.0024 (13)	0.0374 (16)	0.0010 (15)
C4	0.0605 (17)	0.0456 (14)	0.0858 (19)	0.0021 (12)	0.0000 (15)	−0.0082 (13)
C5	0.092 (2)	0.0520 (16)	0.0706 (18)	0.0115 (14)	0.0173 (16)	−0.0169 (13)
C6	0.0689 (17)	0.0553 (15)	0.0634 (15)	0.0096 (13)	0.0281 (13)	−0.0095 (12)
C7	0.0318 (11)	0.0474 (12)	0.0407 (11)	0.0080 (9)	0.0086 (9)	0.0010 (9)
C8	0.0329 (11)	0.0428 (11)	0.0398 (11)	0.0057 (9)	0.0088 (9)	0.0019 (9)
C9	0.0374 (12)	0.0468 (12)	0.0440 (12)	0.0053 (10)	0.0113 (9)	0.0051 (9)
C10	0.0477 (14)	0.0475 (13)	0.0621 (14)	−0.0023 (10)	0.0145 (11)	0.0070 (11)
C11	0.0540 (14)	0.0445 (12)	0.0585 (14)	−0.0030 (11)	0.0088 (11)	−0.0057 (10)
C12	0.0455 (13)	0.0439 (12)	0.0486 (12)	0.0052 (10)	0.0144 (10)	−0.0007 (10)
C13	0.0333 (11)	0.0432 (11)	0.0400 (11)	0.0062 (9)	0.0082 (9)	−0.0002 (9)
C14	0.0421 (12)	0.0473 (12)	0.0479 (12)	0.0091 (10)	0.0154 (10)	0.0005 (10)
C15	0.0400 (12)	0.0503 (13)	0.0560 (14)	0.0094 (10)	0.0192 (10)	0.0031 (10)
C16	0.0498 (13)	0.0598 (14)	0.0421 (12)	0.0112 (11)	0.0199 (10)	−0.0044 (10)
C17	0.0438 (13)	0.0783 (17)	0.0466 (13)	0.0153 (12)	0.0141 (10)	−0.0049 (12)
C18	0.0449 (14)	0.0878 (19)	0.0496 (14)	0.0157 (12)	0.0185 (11)	0.0167 (12)
C19	0.0448 (13)	0.0787 (17)	0.0440 (13)	0.0149 (12)	0.0135 (10)	0.0100 (11)
C20	0.0312 (11)	0.0647 (14)	0.0365 (11)	−0.0034 (10)	0.0129 (9)	0.0008 (10)
C21	0.0422 (12)	0.0505 (13)	0.0413 (12)	0.0065 (10)	0.0194 (10)	0.0049 (9)
C22	0.0505 (14)	0.0544 (14)	0.0616 (15)	0.0082 (11)	0.0077 (12)	0.0006 (11)
C23	0.0472 (16)	0.0771 (19)	0.093 (2)	0.0052 (14)	0.0049 (14)	0.0210 (17)
C24	0.0659 (19)	0.106 (2)	0.090 (2)	0.0389 (18)	0.0401 (18)	0.040 (2)
C25	0.111 (3)	0.084 (2)	0.073 (2)	0.0495 (19)	0.051 (2)	0.0113 (16)
C26	0.0785 (19)	0.0678 (16)	0.0510 (15)	0.0202 (14)	0.0193 (13)	−0.0004 (12)
N1	0.0399 (11)	0.0538 (12)	0.0583 (12)	0.0072 (8)	0.0179 (9)	−0.0006 (9)
N2	0.0348 (10)	0.0452 (10)	0.0495 (10)	0.0071 (8)	0.0137 (8)	−0.0033 (8)
N3	0.0403 (11)	0.0930 (15)	0.0373 (10)	0.0200 (10)	0.0149 (8)	0.0040 (10)
N4	0.0423 (11)	0.0793 (14)	0.0336 (10)	0.0132 (9)	0.0160 (8)	−0.0005 (9)
O1	0.0378 (8)	0.0556 (10)	0.0638 (10)	0.0034 (7)	0.0193 (7)	0.0046 (7)
O2	0.0468 (9)	0.0918 (13)	0.0377 (8)	0.0095 (8)	0.0210 (7)	0.0037 (8)
S1	0.0570 (4)	0.0477 (4)	0.0684 (4)	0.0070 (3)	0.0272 (3)	−0.0110 (3)

### Geometric parameters (Å, °)

**Table:**

C1—C2	1.379 (3)	C15—C16	1.531 (3)
C1—C6	1.389 (3)	C15—H15	0.9800
C1—C7	1.474 (3)	C16—C17	1.508 (3)
C2—C3	1.380 (3)	C16—H16A	0.9700
C2—H2	0.9300	C16—H16B	0.9700
C3—C4	1.371 (4)	C17—N3	1.463 (3)
C3—H3	0.9300	C17—H17A	0.9700
C4—C5	1.361 (4)	C17—H17B	0.9700
C4—H4	0.9300	C18—N3	1.458 (3)
C5—C6	1.375 (4)	C18—C19	1.515 (3)
C5—H5	0.9300	C18—H18A	0.9700
C6—H6	0.9300	C18—H18B	0.9700
C7—N1	1.313 (2)	C19—H19A	0.9700
C7—C8	1.430 (3)	C19—H19B	0.9700
C8—C9	1.346 (3)	C20—O2	1.225 (2)
C8—C13	1.470 (3)	C20—N3	1.355 (3)
C9—O1	1.345 (2)	C20—N4	1.365 (3)
C9—C10	1.484 (3)	C21—C26	1.373 (3)
C10—C11	1.531 (3)	C21—C22	1.384 (3)
C10—H10A	0.9700	C21—N4	1.419 (3)
C10—H10B	0.9700	C22—C23	1.371 (4)
C11—C12	1.506 (3)	C22—H22	0.9300
C11—H11A	0.9700	C23—C24	1.352 (4)
C11—H11B	0.9700	C23—H23	0.9300
C12—C13	1.356 (3)	C24—C25	1.370 (5)
C12—S1	1.720 (2)	C24—H24	0.9300
C13—N2	1.375 (3)	C25—C26	1.393 (4)
C14—N2	1.301 (3)	C25—H25	0.9300
C14—C15	1.498 (3)	C26—H26	0.9300
C14—S1	1.744 (2)	N1—O1	1.413 (2)
C15—C19	1.527 (3)	N4—H4A	0.854 (10)
			
C2—C1—C6	118.0 (2)	C17—C16—H16A	109.3
C2—C1—C7	122.26 (19)	C15—C16—H16A	109.3
C6—C1—C7	119.6 (2)	C17—C16—H16B	109.3
C1—C2—C3	120.6 (2)	C15—C16—H16B	109.3
C1—C2—H2	119.7	H16A—C16—H16B	108.0
C3—C2—H2	119.7	N3—C17—C16	110.11 (19)
C4—C3—C2	120.7 (3)	N3—C17—H17A	109.6
C4—C3—H3	119.7	C16—C17—H17A	109.6
C2—C3—H3	119.7	N3—C17—H17B	109.6
C5—C4—C3	119.1 (2)	C16—C17—H17B	109.6
C5—C4—H4	120.5	H17A—C17—H17B	108.2
C3—C4—H4	120.5	N3—C18—C19	110.86 (19)
C4—C5—C6	121.0 (2)	N3—C18—H18A	109.5
C4—C5—H5	119.5	C19—C18—H18A	109.5
C6—C5—H5	119.5	N3—C18—H18B	109.5
C5—C6—C1	120.6 (3)	C19—C18—H18B	109.5
C5—C6—H6	119.7	H18A—C18—H18B	108.1
C1—C6—H6	119.7	C18—C19—C15	111.3 (2)
N1—C7—C8	110.43 (19)	C18—C19—H19A	109.4
N1—C7—C1	117.71 (18)	C15—C19—H19A	109.4
C8—C7—C1	131.73 (19)	C18—C19—H19B	109.4
C9—C8—C7	104.49 (18)	C15—C19—H19B	109.4
C9—C8—C13	116.91 (19)	H19A—C19—H19B	108.0
C7—C8—C13	138.57 (19)	O2—C20—N3	121.71 (19)
O1—C9—C8	110.89 (18)	O2—C20—N4	121.6 (2)
O1—C9—C10	121.44 (18)	N3—C20—N4	116.71 (18)
C8—C9—C10	127.67 (19)	C26—C21—C22	118.9 (2)
C9—C10—C11	109.03 (18)	C26—C21—N4	123.1 (2)
C9—C10—H10A	109.9	C22—C21—N4	117.95 (19)
C11—C10—H10A	109.9	C23—C22—C21	120.5 (3)
C9—C10—H10B	109.9	C23—C22—H22	119.7
C11—C10—H10B	109.9	C21—C22—H22	119.7
H10A—C10—H10B	108.3	C24—C23—C22	121.1 (3)
C12—C11—C10	111.11 (18)	C24—C23—H23	119.4
C12—C11—H11A	109.4	C22—C23—H23	119.4
C10—C11—H11A	109.4	C23—C24—C25	119.0 (3)
C12—C11—H11B	109.4	C23—C24—H24	120.5
C10—C11—H11B	109.4	C25—C24—H24	120.5
H11A—C11—H11B	108.0	C24—C25—C26	121.0 (3)
C13—C12—C11	124.5 (2)	C24—C25—H25	119.5
C13—C12—S1	108.90 (17)	C26—C25—H25	119.5
C11—C12—S1	126.36 (16)	C21—C26—C25	119.4 (3)
C12—C13—N2	116.70 (18)	C21—C26—H26	120.3
C12—C13—C8	117.74 (19)	C25—C26—H26	120.3
N2—C13—C8	125.54 (18)	C7—N1—O1	106.56 (16)
N2—C14—C15	123.1 (2)	C14—N2—C13	110.80 (18)
N2—C14—S1	113.77 (16)	C20—N3—C18	119.97 (18)
C15—C14—S1	123.14 (16)	C20—N3—C17	127.24 (18)
C14—C15—C19	114.20 (19)	C18—N3—C17	112.65 (18)
C14—C15—C16	110.96 (18)	C20—N4—C21	123.28 (18)
C19—C15—C16	109.26 (17)	C20—N4—H4A	122.1 (17)
C14—C15—H15	107.4	C21—N4—H4A	112.7 (17)
C19—C15—H15	107.4	C9—O1—N1	107.61 (15)
C16—C15—H15	107.4	C12—S1—C14	89.84 (10)
C17—C16—C15	111.60 (19)		
			
C6—C1—C2—C3	−0.6 (4)	C19—C15—C16—C17	−54.2 (3)
C7—C1—C2—C3	−176.5 (2)	C15—C16—C17—N3	56.6 (3)
C1—C2—C3—C4	0.2 (4)	N3—C18—C19—C15	−55.6 (3)
C2—C3—C4—C5	0.6 (4)	C14—C15—C19—C18	178.19 (19)
C3—C4—C5—C6	−1.1 (4)	C16—C15—C19—C18	53.3 (3)
C4—C5—C6—C1	0.7 (4)	C26—C21—C22—C23	−2.8 (4)
C2—C1—C6—C5	0.1 (4)	N4—C21—C22—C23	179.3 (2)
C7—C1—C6—C5	176.1 (2)	C21—C22—C23—C24	0.3 (4)
C2—C1—C7—N1	155.8 (2)	C22—C23—C24—C25	1.8 (4)
C6—C1—C7—N1	−20.0 (3)	C23—C24—C25—C26	−1.3 (5)
C2—C1—C7—C8	−19.6 (3)	C22—C21—C26—C25	3.2 (4)
C6—C1—C7—C8	164.5 (2)	N4—C21—C26—C25	−179.0 (2)
N1—C7—C8—C9	−1.3 (2)	C24—C25—C26—C21	−1.2 (4)
C1—C7—C8—C9	174.4 (2)	C8—C7—N1—O1	0.9 (2)
N1—C7—C8—C13	−178.7 (2)	C1—C7—N1—O1	−175.46 (16)
C1—C7—C8—C13	−3.1 (4)	C15—C14—N2—C13	178.04 (19)
C7—C8—C9—O1	1.2 (2)	S1—C14—N2—C13	0.0 (2)
C13—C8—C9—O1	179.31 (16)	C12—C13—N2—C14	−0.5 (3)
C7—C8—C9—C10	−178.8 (2)	C8—C13—N2—C14	177.82 (19)
C13—C8—C9—C10	−0.7 (3)	O2—C20—N3—C18	−3.7 (3)
O1—C9—C10—C11	−151.80 (19)	N4—C20—N3—C18	175.6 (2)
C8—C9—C10—C11	28.2 (3)	O2—C20—N3—C17	171.6 (2)
C9—C10—C11—C12	−39.1 (2)	N4—C20—N3—C17	−9.1 (4)
C10—C11—C12—C13	30.6 (3)	C19—C18—N3—C20	−125.6 (2)
C10—C11—C12—S1	−155.19 (17)	C19—C18—N3—C17	58.5 (3)
C11—C12—C13—N2	175.8 (2)	C16—C17—N3—C20	125.6 (2)
S1—C12—C13—N2	0.7 (2)	C16—C17—N3—C18	−58.8 (3)
C11—C12—C13—C8	−2.7 (3)	O2—C20—N4—C21	−14.7 (3)
S1—C12—C13—C8	−177.74 (15)	N3—C20—N4—C21	166.0 (2)
C9—C8—C13—C12	−13.7 (3)	C26—C21—N4—C20	48.3 (3)
C7—C8—C13—C12	163.5 (2)	C22—C21—N4—C20	−133.9 (2)
C9—C8—C13—N2	168.03 (19)	C8—C9—O1—N1	−0.7 (2)
C7—C8—C13—N2	−14.8 (4)	C10—C9—O1—N1	179.28 (18)
N2—C14—C15—C19	149.7 (2)	C7—N1—O1—C9	−0.1 (2)
S1—C14—C15—C19	−32.5 (3)	C13—C12—S1—C14	−0.54 (16)
N2—C14—C15—C16	−86.3 (3)	C11—C12—S1—C14	−175.5 (2)
S1—C14—C15—C16	91.5 (2)	N2—C14—S1—C12	0.30 (17)
C14—C15—C16—C17	179.03 (18)	C15—C14—S1—C12	−177.70 (19)

### Hydrogen-bond geometry (Å, °)

**Table:**

*D*—H···*A*	*D*—H	H···*A*	*D*···*A*	*D*—H···*A*
C17—H17A···O2^i^	0.97	2.40	3.353 (3)	167
N4—H4A···O2^i^	0.85 (1)	2.15 (1)	2.976 (2)	164 (2)

Symmetry codes: (i) *x*, −*y*+1/2, *z*−1/2.

## References

[bb1] Bond, J. W., Hachisu, Y., Matsuura, T. & Suzuk, K. (2003). *Org. Lett.* **5**, 319–394. 10.1021/ol027283f 12583726

[bb2] Bruker (2001). *SMART* and *SAINT* Bruker AXS Inc., Madison, Wisconsin, USA.

[bb3] Duff, S. M. G., Chen, Y.-C. S., Fabbri, B. J., Yalamanchili, G., Hamper, B. C., Walker, D. M., Brookfiled, F. A., Boyd, E. A., Ashton, M. R., Yarnold, C. J. & Cajacob, C. A. (2007). *Pestic. Biochem. Physiol.* **88**, 1–3.

[bb4] Hu, D.-J., Liu, S.-F., Huang, T.-H., Tu, H.-Y. & Zhang, A.-D. (2009). *Molecules*, **14**, 1288–1303. 10.3390/molecules14031288 PMC6253765 19325524

[bb5] Sheldrick, G. M. (1996). *SADABS* University of Göttingen, Germany.

[bb6] Sheldrick, G. M. (2008). *Acta Cryst.* A**64**, 112–122. 10.1107/S0108767307043930 18156677

[bb7] Spek, A. L. (2009). *Acta Cryst.* D**65**, 148–155. 10.1107/S090744490804362X PMC2631630 19171970

